# Providing solution to drowning: a scoping review on epidemiological studies

**Published:** 2022-12

**Authors:** Seyed Hossein Mousavi, Somayeh Panahi

**Affiliations:** ^*a*^ Vice-Chancellor for Research, Bushehr University of Medical Sciences, Bushehr, Iran.; ^*b*^ Medical Library and Information Science Department, Health Information Technology Research Center, Isfahan University of Medical Sciences, Isfahan, Iran.

## Abstract

**Background:**

Drowning is the third cause of worldwide unintentional death in public health. This study aimed to extract the main components from epidemiological studies (investigating demographic differences, autopsy findings, and factors influencing the drowning of victims in each geographic region) for further development of prevention research.

**Methods:**

The scoping review was based on the Arksey and O’Malley framework. First, the research question was specified. Second, relevant literature was identified, and relevant studies were selected. Then, the data were mapped out. The results were summarized, synthesized, and reported. Finally, expert consultation was conducted. The articles published from the beginning to 2022 which were indexed in PubMed and Web of Science were retrieved using keywords referring to “drowning”. Besides, VOSviewer software was used to explore more data. Subsequently, 64 documents were screened.

**Results:**

Death due to drowning is a common event in the world. It occurs firstly due to incautiousness and also due to various conditions like suicide, alcohol consumption, accident, and murder. Low- and middle-income countries, residents of rural areas, people with lower literacy levels, children and teenagers with mothers unfamiliar with swimming skills, and people with poor swimming skills are the most damaged.

**Conclusions:**

Teaching drowning prevention is recommended which results in disaster health literacy, a higher understanding of risk, social preventive measures, and community-based participation in all dimensions of the disaster management cycle (prevention, mitigation, preparedness, response, and recovery). The courses can be included in the educational system and other general education programs. Since children are among the groups at risk, it is recommended that health policymakers make the necessary arrangements for tele-education, especially for mothers, to ensure the effectiveness and level of this type of literacy. Moreover, it is suggested to establish an early warning system (EWS) and improve health literacy for increasing awareness and integrating it into this system.

**Keywords:**

Drowning, Epidemiology, Education, Literacy, Disasters

